# Improving measurement-based care implementation in adult ambulatory psychiatry: a virtual focus group interview with multidisciplinary healthcare professionals

**DOI:** 10.1186/s12913-023-09202-3

**Published:** 2023-04-26

**Authors:** Hayoung Ko, Alyssa J. Gatto, Sydney B. Jones, Virginia C. O’Brien, Robert S. McNamara, Martha M. Tenzer, Hunter D. Sharp, Anita S. Kablinger, Lee D. Cooper

**Affiliations:** 1 Department of Psychology, Virginia Polytechnic Institute and State University, Blacksburg, VA USA; 2 Department of Psychiatry and Human Behavior, Brown University, Providence, RI USA; 3 Department of Psychiatry and Behavioral Medicine, Virginia Tech Carilion School of Medicine, Roanoke, Virginia USA; 4 Health Analytics Research Team, Carilion Clinic, Roanoke, Virginia USA

**Keywords:** Measurement-based care, Implementation, Focus Groups, Psychiatry, Healthcare, Multidisciplinary

## Abstract

**Background:**

Measurement-Based Care (MBC) is an evidence-based practice shown to enhance patient care. Despite being efficacious, MBC is not commonly used in practice. While barriers and facilitators of MBC implementation have been described in the literature, the type of clinicians and populations studied vary widely, even within the same practice setting. The current study aims to improve MBC implementation in adult ambulatory psychiatry by conducting focus group interviews while utilizing a novel virtual brainwriting premortem method.

**Methods:**

Semi-structured focus group interviews were conducted with clinicians (*n* = 18) and staff (*n* = 7) to identify their current attitudes, facilitators, and barriers of MBC implementation in their healthcare setting. Virtual video-conferencing software was used to conduct focus groups, and based on transcribed verbatin, emergent barriers/facilitators and four themes were identified. Mixed methods approach was utilized for this study. Specifically, qualitative data was aggregated and re-coded separately by three doctoral-level coders. Quantitative analyses were conducted from a follow-up questionnaire surveying clinician attitudes and satisfaction with MBC.

**Results:**

The clinician and staff focus groups resulted in 291 and 91 unique codes, respectively. While clinicians identified a similar number of barriers (40.9%) and facilitators (44.3%), staff identified more barriers (67%) than facilitators (24.7%) for MBC. Four themes emerged from the analysis; (1) a description of current status/neutral opinion on MBC; (2) positive themes that include benefits of MBC, facilitators, enablers, or reasons on why they conduct MBC in their practice, (3) negative themes that include barriers or issues that hinder them from incorporating MBC into their practice, and (4) requests and suggestions for future MBC implementation. Both participant groups raised more negative themes highlighting critical challenges to MBC implementation than positive themes. The follow-up questionnaire regarding MBC attitudes showed the areas that clinicians emphasized the most and the least in their clinical practice.

**Conclusion:**

The virtual brainwriting premortem focus groups provided critical information on the shortcomings and strengths of MBC in adult ambulatory psychiatry. Our findings underscore implementation challenges in healthcare settings and provide insight for both research and clinical practice in mental health fields. The barriers and facilitators identified in this study can inform future training to increase sustainability and better integrate MBC with positive downstream outcomes in patient care.

**Supplementary Information:**

The online version contains supplementary material available at 10.1186/s12913-023-09202-3.

## Background

The practice of regularly evaluating one’s mental and emotional states in search of patterns or trends is a common informal practice that has long been found to facilitate increased social and emotional improvement [1, 2]. When formally conducted in clinical settings, the utilization of Patient-Reported Outcome Measures (PROMs) and their results to drive clinical decision making is known as Measurement-Based Care (MBC) [3]. MBC has been investigated for its added clinical value and utility, and research findings repeatedly indicate the multitude of benefits to psychiatric patients and clinicians alike [4–8].

Though research is supportive of MBC as an evidence-based practice (EBP) for mental and behavioral healthcare, the literature also suggests significant barriers to successful implementation [4, 9–12]. Providers are more than capable of administering and interpreting psychosocial assessments; however, MBC has proven challenging to integrate into care [13–16]. Previous studies have found that less than 20% of clinicians or providers regularly administer MBC to their patients despite the availability of a wide range of validated measures that reliably reflects changes in symptom severity [17, 18]. In addition, over 60% of clinicians have never used a Measurement Feedback System (MFS) [19, 20], a web- and mobile-based electronic platform that assists administration, scoring, and interpretation of multiple measures to facilitate MBC. Clinicians have regularly identified barriers to routine MBC implementation (e.g., lengthy assessments), which affects time and patient commitment, and personal endorsement of symptoms [21–23].

There are hundreds of dissemination and implementation frameworks intended to guide the integration of an EBP across settings [5, 24, 25]. To enhance the integration of MBC, this study narrows on the Practical, Robust Implementation and Sustainability Model (PRISM) [26] as the guiding framework. The PRISM model builds off pre-existing models (i.e., Diffusion of Innovations, the Chronic Care Model; Model for Improvement) and merges with the RE-AIM framework [27] to act as a critical tool to translate research into practice. The model itself involves several domains: intervention (e.g., new EBP), recipients (individuals who receive or implement the EBP), external environment (e.g., the continuing pandemic), and implementation (e.g., actual use of the new technique) and sustainability infrastructure (e.g., supports put in place by a hospital to facilitate and maintain the new procedure). These major domains provide structure for the broader context and identify critical stakeholders who inform program implementation and dissemination. The program can then be evaluated within the RE-AIM framework to evaluate Reach (e.g., equitably implementing treatment to target population), Effectiveness (e.g., impact of intervention in improving clinical outcomes), Adoption (e.g., recipient’s commitment level/status), Implementation (e.g., fidelity to intervention delivery), and Maintenance (e.g., long-term intervention sustainability). Previous research integrating PRISM has shown the most successful implementation occurs when three or more domains (e.g., intervention, recipients, external environment) are considered along with at least one element within these domains (e.g., intervention (strength of the evidence base), recipient (management support and communication), or implementation (adaptable protocols and procedures) [28–32]. PRISM promotes identifying and documenting key factors or leverage points at multiple levels of internal and external influence for implementation success.

In a healthcare setting, it is critical to examine the organizational and structural barriers in conjunction with psychological barriers to integrate an effective EBP such as MBC. There are many approaches to understand ambivalence to MBC that include surveying or interviewing stakeholders involved in the intervention-organizational perspective and recipients-organizational characteristics domains within the PRISM implementation model [26]. From this context, MBC (intervention) is received by patients (recipients) and implemented by clinicians and staff (recipients) in mental healthcare settings within the broader context of a global pandemic (external environment). At the same time, a sustainability infrastructure needs to be created to ensure systematic support and ongoing consultation by the healthcare setting to facilitate utilization of MBC. It is necessary to take multiple approaches to understand an organization’s current infrastructure supporting an EPB. As such, we sought to better understand MBC supports currently embedded within the organization to identify changes that can be made to improve adoption by the recipients. In addition to the team’s expertise functioning within this institution, one promising method to disentangle the organizational perspective is using the Brainwriting Premortem Method (BPM) to hear directly from those responsible for delivering and assisting MBC [33]. The BPM was designed to identify failures and limitations a-priori to ensure better implementation and dissemination of EBPs. BPM uses a group brainstorming approach with a flexible interviewing style to receive input from multiple participants at once. This method supports participants’ ability to share ideas simultaneously and to agree or counter other ideas, thus ensuring more rapid and efficient data collection when compared to traditional focus groups. Utilizing the BPM can assist in better MBC integration with downstream improvements in health system performance and ultimately health outcomes. This method has been successfully utilized as an important tool guiding implementation; specifically, to inform emotion regulation prevention program development with college students [34], improve care coordination for rural veterans [35], to inform the scale-up of a nursing intervention [36], improving electronic health records with user-centered design [37], inform adoption of technology in higher education settings in South Africa [38], and assess suicide-specific training for mental health providers of active-duty military personnel experiences [39].

### Present study

It is apparent that standardized training is necessary for successful implementation of MBC [40, 41], though which aspects are critical for successful and relevant training have yet to be identified. To date, there are no studies that have implemented BPM focus groups in psychiatric settings, which include multidisciplinary healthcare professionals, to improve the effectiveness of MBC implementation. Instead, the literature delineates a plethora of barriers with intermittent success stories. As such, this study is focused on identifying the obstacles as well as facilitators to efficient implementation prior to any formalized training. This study applies the PRISM framework to evaluate the recipients, intervention, and external environment of MBC to create an implementation and sustainability infrastructure which ensures effective use of MBC and informs training efforts. Using a virtual adaptation of the BPM, the current study conducted focus groups to assess the understanding and opinions of MBC implementation by clinicians and staff, who have been involved in incorporating MBC into their usual care. Furthermore, the utility and feasibility of a virtual setting for conducting the focus groups was evaluated. Together, using the mixed method design, data was organized within the PRISM framework to inform current MBC implementation and build a plan for sustainability.

## Methods

### Participants

Two samples of participants (*N* = 25) were recruited from the Department of Psychiatry and Behavioral Medicine’s (PBM) outpatient clinic in a US regional hospital, including a clinician sample of 18 (72%) and a staff sample of 7 (28%). This sample was representative of the majority of staff and clinicians in PBM. PBM rolled out a new MFS in April 2019 prior to conducting focus groups. Additionally, the department attempted a brief training 16–18 months before focus groups to provide education and delineate the purpose of the MFS during provider meetings, which included new workflows. Educational materials included ways to access the MFS, a review of selected PROMs, and scoring guidelines. Faculty champions of MBC were available for assistance as needed as this informal training was primarily focused on utilization of the new MFS and workflow changes. From a patient perspective, once the patients completed the initial bundle of PROMs, they received and completed the major PROMs on a regular basis. Demographic information is presented in Table 1. Data collection was approved by the Carilion Clinic Institutional Review Board (IRB #20-1065). The participation was voluntary and recommended by the clinic as part of quality improvement and the participants’ involvement in the study was anonymous to their supervisor/administrator to ensure voluntary participation.

**Table 1:** Demographic characteristics of clinicians and staff

	Clinicians (n = 18)		Staff (n = 7)
Age		Age	
25–35	3 (16.7%)	25–35	1 (14.3%)
36–45	7 (38.9%)	36–45	2 (28.6%)
46–55	6 (33.3%)	46–55	3 (42.9%)
> 55	2 (11.1%)	> 55	1 (14.3%)
Gender		Gender	
Female	9 (50.0%)	Female	7 (100.0%)
Male Unspecified	7 (38.9%) 2 (11.1%)	Male Unspecified	0 (0.0%) 0 (0.0%)
Length of time employed in psychiatric field	7.44 (SD = 11.97)		
Length of time employed at this institution		Length of time employed at this institution	
< 1 year	1 (5.6%)	< 1 year	1 (14.3%)
1–2 years	4 (22.2%)	1–2 years	2 (28.6%)
3–5 years	8 (44.4%)	3–5 years	1 (14.3%)
> 5 years	5 (27.8%)	> 5 years	3 (42.9%)
Degrees obtained		Degrees obtained	
MD	10 (55.5%)	Some college, no degree	2 (28.6%)
NP	5 (27.8%)	Associate’s degree	2 (28.6%)
PhD	1 (5.6%)	Bachelor’s degree	1 (14.3%)
LCSW	2 (11.1%)	Master’s degree	0 (0.0%)
Other	0 (0.0%)	Doctorate degree	0 (0.0%)
		Other	2 (28.6%)
Primary duties/activities regarding MBC	Provide detailed explanation on MBC including overall procedure and benefits to the patients’ intervention; Collaboratively review PROM results with patients in session; Utilize and integrate PROM results to inform and/or adjust therapeutic intervention	Primary duties/activities regarding MBC	Provide initial introduction of MBC and MFS; Explain the timeline for PROM completion; Facilitate completion of PROM if not conducted prior to the appointment; Address patients’ technical difficulties or challenges as needed

#### Clinician

Inclusion criteria for the clinician group included providers actively seeing patients at the PBM outpatient clinic. Participants were recruited via personalized emails. The sample (*N* = 18) was distributed between female (*n* = 9; 50.0%), male (*n* = 7; 38.9%), and unspecified (*n* = 2; 11.1%) clinicians, and their age ranges are as follows: 25–35 (*n* = 3, 16.7%), 36–45 (*n* = 7, 38.9%), 46–55 (*n* = 6, 33.3%), and > 55 (*n* = 2, 11.1%). All participants were full-time clinicians, and their credentials were as follows: MD (*n* = 10, 55.5%), NP (*n* = 5, 27.8%), PhD (*n* = 1, 5.6%), and LCSW (*n* = 2, 11.1%). Participants were employed at this institution from < 1 year (*n* = 1, 5.6%), 1–2 years (*n* = 4, 22.2%), 3–5 years (*n* = 8, 44.4%), to > 5 years (*n* = 5, 27.8%). The participants’ average length of time in practice was 7.44 (*SD* = 11.97) years. Regarding MBC implementation, clinicians’ were responsible for the following: providing detailed explanation of MBC including overall procedure and benefits to the patients’ intervention; collaboratively reviewing PROM results with patients in session; and utilization and integration of PROM results to inform therapeutic intervention.

#### Staff

Eligible staff were full-time employees experienced and involved in the process of MBC with patients, recruited from the same outpatient clinic via personalized emails. Participants were all female (*N* = 7, 100.0%) with ages ranging from 25 to 35 (*n* = 1, 14.3%), 36–45 (*n* = 2, 28.6%), 46–55 (*n* = 3, 42.9%), to > 55 (*n* = 1, 14.3%). Their duration of employment at this outpatient clinic ranged from < 1 year (*n* = 1, 14.3%), 1–2 years (*n* = 2, 28.6%), 3–5 years (*n* = 1, 14.3%), to > 5 years (*n* = 3, 42.9%). The staff participants’ educational level was some college-no degree (n = 2, 28.6%), associate’s degree (*n* = 2, 28.6%), bachelor’s degree (*n* = 1, 14.3%), and other (*n* = 2, 28.6%). Staff roles in MBC implementation were as follows: Providing initial introduction of MBC and MFS; explaining the timeline for PROM completion (e.g., prior to initial appointment and then monthly); facilitating completion of PROM if not conducted prior to the appointment; and addressing patients’ technical difficulties or challenges as needed.

### Materials

#### Zoom

In this study, all focus groups were conducted via HIPAA-compliant Zoom video conferencing. Focus group components and visuals were digitally presented to the participants.

#### Google Docs

All content was presented via Google Docs to participants. Participants provided their anonymized responses to questions by typing their answers directly onto the Google Doc. Participants were also allowed to provide their responses verbally, especially if they were having technical issues or visual impairments, and in this case, the research assistant recorded their answers.

### Measurement

#### Qualitative Data

The current study developed focus group questionnaires in the context of PRISM in order to grasp both clinician and staff attitudes towards specific PRISM components (e.g., intervention itself, external environment, recipients’ perspective). For the clinician focus groups, 21 questions were created regarding their attitudes and usage of MBC and PROMs including: reason for using PROMs, how they are utilizing MBC and PROMs in their practice, evaluation of MBC implementation in their clinic, feedback from their patients regarding MBC and PROMs completion, and barriers/supports to engaging in MBC (see Appendix A). For the staff focus groups, there were 17 questions including their overall understanding of the MBC process, feedback regarding the MFS, barriers/supports in conducting MBC, and thoughts about potential MBC training (see Appendix B).

#### Clinician attitudes toward and satisfaction with MBC

An additional evaluation form (containing 7 items) was created to fully capture clinicians’ attitudes toward MBC and their satisfaction with the MFS currently utilized in the PBM clinic. Specifically, questions assessed clinicians’ perception of the usefulness of MBC, clinician utilization (i.e., explaining the purpose of regular monitoring to their patients/ providing feedback about PROMs to their patients), the overall attitude towards MBC (i.e., likeliness to continue incorporating MBC into their clinical practice) and clinicians’ opinions of the MFS they were using (i.e., whether the MFS interface is easy to navigate). This measure was administered one time after focus group completion. Attitudes and satisfaction ratings were based on a 5-point scale rating from 1 (e.g., extremely negative) to 5 (e.g., extremely positive).

### Procedure

Participants attended a 45-minute to 1-hour online session via videoconferencing software. Virtual focus groups are an unexpected side effect of the COVID-19 pandemic that have changed the accessibility and methods for data collection. A virtual setting can be adaptive in that it allows for participants to join without travel burdens or clinical absences and has shown itself to be widely and freely accessible. For the current study, the use of the BPM was unimpeded by the virtual focus group setting as participants were largely able to access the free public software used to share ideas both verbally, as well as in writing. Clinicians and staff joined focus groups from their office during their work shift. At the start of the session, participants and leaders introduced themselves while engaging in collaborative rule-setting. Participants were sent a Google Doc link where they were instructed to sign out of Google accounts to ensure anonymity and to providing their responses, while still being able to ask questions or communicate with the focus group leaders as needed. Doctoral student leaders, who had no previous relationship with any participants, guided clinicians and staff through a standard set of questions. Specifically, the participants typed their responses to open ended questions into a Google Doc form while guided by the focus group leader. The focus group leader introduced questions and assisted participants with further queries about each question regarding MBC practice. While the participants were writing their own answer, they were also able to see what other participants were writing or responding to the same question, facilitating further discussion, as the participants answered the questions concomitantly. Participants expanded their responses in a written form in the same document, or at times, verbally expressed additional thoughts. In this case, the research assistant recorded their verbatim responses and these were incorporated into the final data. Though common technical difficulties (e.g., poor or weak internet connection, audio/video adjustment) occurred during the execution of these focus groups, focus group leaders were able to successfully retrieve meaningful information from participants. When participants had continuous difficulties engaging with the virtual platform (e.g., participants with visual impairments, technical difficulties), BPM was implemented with flexibility (e.g., participants verbally expressed their opinion rather than typing).

### Data analyses

This study utilized a mixed-method design and the analytic plans are as follows.

#### Qualitative Analysis

Clinician and staff participants’ raw answers to pre-established questions during the online sessions resulted in hundreds of individual responses which were aggregated into unique codes encompassing similar themes. For example, when asked what may cause problems in future proposed MBC training, clinicians provided different responses including “Training takes [too] much time”, and “Complexity of training could be problematic” which were then aggregated into one consolidated theme, “time burden for training.” This theme, “time burden for training,” was then classified as both pertaining in major part to clinician concerns as well as to patient experience. When totaling the initial number of codes, this method of double, or even triple coding some responses generates a larger number of codes than there are responses. Through interrater collaboration and review, these codes were later designated as pertaining primarily to one most-relevant group (i.e., patient, clinician, institution, staff, technology).

To determine emergent themes regarding potential facilitators and barriers for the systematic and standardized MBC training, constructivist grounded theory [42] was adopted as a framework for qualitative analysis in this study. This strategy allows topics and concepts to arise naturally rather than being guided by predetermined theoretical frameworks. After the completion of all focus groups, raw interview content from each participant was organized and re-coded by doctoral-level researchers. The verification of data integrity and the data coding process was separately conducted by three coders, and thorough discussion and consensus were used to resolve any disagreements. Once the re-coding was completed, the researchers categorized them in three different ways. First, each response was categorized into barriers, facilitators, or protocol related to the current MBC procedures and implementation. Second, as subcategories of barriers/facilitators/protocol, each response was also categorized into patient-, clinician-, staff-, technology-, and institution-focused, according to the target of comments. Finally, the responses were categorized into four themes including neutral/current status (e.g., if the code is neither positive nor negative but provides information on their current understanding or MBC practice), positive themes (i.e., benefits noted), negative themes (i.e., issues raised), and requests and suggestions for the future training regarding MBC implementation and dissemination within the institution. The current study complied with the Standards for Reporting Qualitative Research (SRQR) [43] in conducting a qualitative analysis and producing a written product for collected data.

#### Quantitative Analysis

Descriptive statistics were used to identify the overall attitude and satisfaction of clinicians towards MBC and the MFS that was utilized in the psychiatry outpatient clinic. The IBM SPSS statistics 27 program was utilized for the descriptive statistics.

## Results

In clinician focus groups, participants’ raw answers to 21 questions resulted in 291 individual codes based on the similarity of raw answers. Likewise, in staff focus groups, participants’ raw answers to 16 questions were aggregated into 97 individual codes.

### Barriers and facilitators

#### Barriers

Clinicians identified 119 (40.9%) barriers among 291 total codes to implementing MBC in their clinical practice. These barriers were categorized into 6 categories: patient (*n* = 61, 51.3%), clinician (*n* = 33, 27.7%), staff (*n* = 4, 3.4%), technology (*n* = 17, 14.3%), institution (*n* = 2, 1.7%), and N/A (*n* = 2, 1.7%; see Table 2). Of these, the patient related barriers were most frequently identified. Staff also identified 65 (67%) barriers among 97 total codes which were also categorized into 5 categories of which technology-related barriers were the most popular (*n* = 24, 36.9%), followed by patient (*n* = 16, 24.6%), staff (*n* = 11, 16.9%), clinician (*n* = 10, 15.4%), and institution (*n* = 4, 6.2%).

**Table 2:** Responses aggregated by targeted focus of comments from clinicians and staff

**Barrier**	**Focus Area**	**Clinician**	**Staff**
	Client	61 (51.3%)	16 (24.6%)
	Clinician	33 (27.7%)	10 (15.4%)
	Staff	4 (3.4%)	11 (16.9%)
	Technology	17 (14.3%)	24 (36.9%)
	Institution	2 (1.7%)	4 (6.2%)
	N/A	2 (1.7%)	0 (0.0%)
	Total	119 (40.9%)	65 (67%)
**Facilitator**	**Focus Area**	**Clinician**	**Staff**
	Client	58 (45.0%)	4 (16.7%)
	Clinician	56 (43.4%)	3 (12.5%)
	Staff	3 (2.3%)	8 (33.3%)
	Technology	8 (6.2%)	4 (16.7%)
	Institution	1 (0.8%)	5 (20.8%)
	N/A	2 (0.8%)	0 (0.0%)
	Total	129 (44.3%)	24 (24.7%)
**Protocol**	**Focus Area**	**Clinician**	**Staff**
	Client	2 (18.2%)	0 (0.0%)
	Clinician	5 (45.5%)	1 (16.7%)
	Staff	0 (0.0%)	3 (50.0%)
	Technology	0 (0.0%)	1(16.7%)
	Institution	0 (0.0%)	1(16.7%)
	N/A	4 (36.4%)	0 (0.0%)
	Total	11 (3.8%)	6 (6.2%)
**N/A**		32 (11.0%)	2 (2.1%)
**Total**		291 (100.0%)	97 (100.0%)

#### Facilitators

Clinicians noted 129 (44.3%) facilitators to the MBC process among 291 total codes. Most notable were patient-related facilitators (*n* = 58, 45.0%), followed by those pertaining to clinician (*n* = 56, 43.4%), technology (*n* = 8, 6.2%), staff (*n* = 3, 2.3%), and institution (*n* = 1, 0.8%). In contrast, staff identified 24 (24.7%) facilitators among 97 total codes, which was a smaller percentage compared to the clinicians’ report of facilitators. Staff-related facilitators were the most commonly noted (*n* = 8, 33.3%), followed by institution (*n* = 5, 20.8%), technology (*n* = 4, 16.7%), patient (*n* = 4, 16.7%), and clinician (*n* = 3, 12.5%).

#### Protocol

Individual codes were categorized as ‘protocol’ if the contents were related to the current process and procedure regarding MBC implementation as mandated by institutional protocol. Clinicians noted 11 protocol codes among 291 total codes, and the most prevalent focus area was clinician (n = 5, 45.5%), followed by patient (n = 2, 18.2%). Staff noted 6 protocol codes among 97 individual codes.

### Main qualitative themes

#### Clinicians focus groups

The clinicians focus group resulted in 291 individual and unique statements that were again, categorized into four main themes including positive, neutral/current status, negative, and requests/suggestions, based on the specific contents of each code. The list of all four themes and sub-themes are presented in Table 3. Among the four themes, the frequency and percentage of individually coded negative themes (i.e., issues raised; 49.5%) was greater than other themes including positive themes (i.e., benefits noted; 21.3%), neutral/current status (16.8%), and requests/suggestions (10.7%). Among negative themes, the most prevalent sub-category was related to difficulty utilizing MBC in certain types of patients (7.9%), followed by patient non-adherence (7.6%), patient’s poor understanding of MBC (7.0%), and that the clinician does not use PROMs but instead uses other information (6.9%; see Table 3 for more sub-categories). Within the positive theme, the most common reports were an overall positive view of MBC (12.0%) that included specific codes such as PROMs help with intake, diagnosis, and clinical insights, certain patients are comfortable and good candidates for PROM use, and PROMs are beneficial, good, and correlate accurately to patients’ feelings. The second most prevalent positive sub-category was patient/clinician review of the results and having a discussion (7.6%), followed by patient’s positive attitude toward MBC (1.7%). Clinicians also reported neutral themes that reflected their current status regarding MBC implementation. This category included codes such as utilize PROMs to guide, track, and monitor treatment and symptoms (7.6%), report on useful (5.8%) and non-useful (1.0%) PROMs, and reports of no feedback, complaint, or noticeable trend from patients in PROM utilization (2.4%). The least reported theme was requests and suggestions (10.7%) including suggestions for future MBC training (4.8%), request for improved implementation/standardization and training/support for the current MBC (4.5%), and request for MBC training for patients (1.4%).

**Table 3:** Themes raised from the clinician focus groups

Category	Individual themes	Frequency of individual codes	Percent	Frequency of clinicians’ answers	Frequency of focus groups (range 1–5)
Neutral/Current Status	Utilize PROMs to Guide/ Track/ Monitor treatment and symptoms No feedback/complaint or no noticeable trend from patients in PROM utilization Report on useful PROMs Report on non-useful PROMs	22 7 17 3	7.6 2.4 5.8 1.0	50 13 51 3	5 2 2 4 1
Positive themes (Benefits noted)	Patient/Clinician review the results and have discussion Patients positive attitude toward MBC - patients like collaborative work on PROMs Positive view of MBC ° PROMs help with intake, diagnosis, and clinical insights ° Certain patients are comfortable/ good candidates for PROM use ° PROMS are beneficial/ good/ correlate accurately to client’s feelings	22 5 8 14 13	7.6 1.7 2.7 4.8 4.5	50 6 11 21 33	5 1 2 1 3
Negative themes (Issues raised)	Negative view of current MBC ° clinician burden/ clinical inconvenience ° Time constraints issue ° PROMs are not broadly useful Negative view of MBC training - clinician burden/clinical inconvenience Patient non-adherence Patients’ lack of understanding of MBC Patients’ negative attitude towards MBC and OWL Lack of designated MBC/Tech staff Chart integration issue COVID/Telehealth barrier Difficulty to utilize MBC in certain types of patients Inappropriateness of PROMs for clients with high suicidality Discrepancies exist between PROMs and Interview reflect inaccuracy Does not use PROM/ Use other information	3 14 7 4 22 2 6 14 5 4 23 12 8 20	1 4.8 2.4 1.4 7.6 0.7 2.1 4.8 1.7 1.4 7.9 4.1 2.7 6.9	9 40 8 9 48 3 10 29 12 4 50 12 26 47	2 4 1 3 4 1 2 3 3 1 3 1 3 3
Requests & Suggestions	Request for improved implementation/standardization and training/support for MBC Request for MBC training for clients Suggestions for future MBC training	13 4 14	4.5 1.4 4.8	33 5 22	3 2 3
N/A		5	1.7	10	3
Total		291	100.00		

#### Staff focus groups

A total of 97 individual staff responses were also categorized into four main themes. Overall, staff focus groups raised more negative themes (staff focus group: 62.9%; clinician focus group: 49.5%) and fewer positive themes (staff focus group: 9.3%; clinician focus group: 21.3%) compared to the clinician focus groups. Among negative themes, staff raised the most issues regarding patients’ negative attitudes toward MBC and MFS (15.5%), followed by technology issues (10.3%), difficulty regarding virtual visits (7.2%), and patient non-adherence (7.2%). Neutral/current status codes accounted for 10.3% of all reported individual themes. The staff mostly reported their current understanding and their duty regarding MBC processes. Requests for MBC training, more support, and suggestions for their preferred training module accounted for 10.3% of staff feedback as well. Positive comments and benefits were the least reported themes among all four categories, and they accounted for 9.3% of all reported codes. Only 4.1% of codes were related to the staff’s positive attitude toward the current MBC process and MFS, and 5.2% accounted for the staffs’ positive attitude towards future MBC training (see Table 4).

**Table 4:** Themes raised from the staff focus groups

Category	Individual themes	Frequency of individual codes	Percent	Frequency of staff’s answers	Frequency of focus groups (range 1–2)
Neutral/Current Status	Staff’s current understanding and duty regarding MBC	10	10.3	11	1
Positive themes (Benefits noted)	Positive attitudes toward OWL and current MBC Positive attitudes toward MBC training	4 5	4.1 5.2	7 8	1 1
Negative themes (Issues raised)	Negative attitudes toward current MBC - MBC is a burden Technology issue Lack of designated MBC staff Patient non-adherence Patients lack of understanding of MBC Patients’ negative attitude towards MBC and OWL Virtual visit difficulty (telehealth barrier) Availability of iPad and related attitudes Chart Integration Issue Limited # of clinicians go through MBC together with their patients Negative attitudes toward MBC training	1 10 4 7 4 15 7 5 5 1 2	1 10.3 4.1 7.2 4.1 15.5 7.2 5.2 5.2 1 2.1	1 22 5 16 5 31 10 13 6 1 6	1 2 1 2 2 2 2 2 2 1 2
Requests & Suggestions	Request for MBC training/more support and suggestion for preferred training module	10	10.3	15	2
N/A		7	7.2	12	2
Total		97	100.00		

### Clinician attitudes and satisfaction

After conducting all five sessions of clinician focus groups, all enrolled clinicians who participated in the clinician focus groups were asked to complete the follow-up survey. Twelve of the eighteen clinicians completed this survey, and clinicians’ ratings are presented in Table 5. Clinicians rated the importance of regular assessment of their patient’s symptoms as well as the explanation of the purpose of regular monitoring to their patients most highly, followed by their likelihood to continuously incorporate MBC into their clinical practice. On the other hand, clinicians rated how often the MBC results are used to inform their clinical decision-making the lowest.

**Table 5:** Clinician attitudes and satisfaction regarding MBC (n = 12)

Question	*Mean*	*SD*	*Range* *1–5*
I feel that MBC is helping my patients become more aware of their symptoms.	3.08	0.29	3–4
I feel that assessing my patients’ symptoms regularly is important to clinical practice.	3.75	0.45	3–4
I feel that the Owl Insights interface is easy to navigate.	3.00	1.04	1–4
I explain the purpose of regular monitoring to my patients.	3.75	0.45	3–4
In general, I provide feedback to my patients about their PROM measures.	3.00	0.85	2–4
How often do you use MBC results to inform clinical decision making?	2.83	0.83	2–4
How likely are you to continue to incorporate MBC into your clinical practice?	3.5	0.67	2–4
***Total***	3.25	0.79	

*Note.* Higher score means more positive attitudes and satisfaction toward MBC

## Discussion

Measurement-Based Care (MBC) is an evidence-based practice that has demonstrated capability to enhance mental and behavioral health care when appropriately implemented. This study utilized the Brainwriting Premortem Method (BPM) [33] to collect data on the current status, opinions, and clinicians’ understanding and attitudes of MBC in an adult outpatient psychiatry setting to understand barriers and facilitators of MBC within the PRISM framework [26]. Virtual focus groups were intended to engage stakeholders and adjust the implementation process in a setting where rapid implementation was conducted without formalized training. The researchers aimed to receive feedback from multidisciplinary professionals, including clinicians and staff, to further the development of MBC training and systematically re-implement MBC at a departmental level. A virtual BPM was born out of the limitations of COVID-19, and this study provides a unique opportunity to determine the successes and limitations of translating this method to a virtual platform while maintaining protective pandemic precautions. The BPM was successful in engaging participants from each focus group and retrieved meaningful information from all participants. Participants’ active engagement in focus groups resulted in 291 individual codes from clinicians and 97 from staff, based on the similarity of their original responses. There were several benefits of virtual focus groups. First, the virtual BPM allowed participants to share their ideas without a heavy time burden, considering travel was not required to participate [44, 45]. Although unexpected technical difficulties or disabilities interfered with full anonymity, participants still provided rich and descriptive responses to each question. Considering that the primary purpose of the BPM was to facilitate an open discussion in an environment that can mitigate psychological burden when participants disagree with others’ opinions, this virtual BPM was successful in gathering various responses, opinions, and attitudes on MBC from different clinicians and staff, considering the wide range of response topics and themes arose. Additionally, clinic leadership personnel were not part of the focus groups, and participants were assured anonymity, especially to their leaders, with the goal of providing as much openness as possible during the focus groups. For the researchers, the use of Google Docs for the virtual BPM reduced the time burden by decreasing the manual labor of transcribing focus group participants’ responses. Despite instances when participants could not type (e.g., network disconnections, disabilities), most provided clear responses in sentences, phrases, or words, which facilitated easy data organization once the focus groups were completed.

On the other hand, there were limitations of virtual BPM. This method was restricted in its ability to retrieve the same amount of information from each participant. Although participants answered most questions, this study did not provide strict guidelines for participation (e.g., each participant should answer all questions, each participant can only answer in less than 3 sentences for each question). This flexibility may have resulted in a difference in response quantity per participant, since their engagement level (i.e., number of verbal/written responses) varied.

When the responses were divided into barrier, facilitator, and protocol categories, the ratio differed by clinicians and staff. Although barriers accounted for a significant portion of clinician responses, there was more of a balance between barriers and facilitators noted for the clinicians as compared to the staff responses. Despite these barriers, as recipients of MBC, clinicians appear aligned with the “shared goal and cooperation” of implementing MBC, implying that the clinicians were tuned in to both barriers and facilitators of conducting and incorporating MBC into their practice and interventions. These results also align with the quantitative analysis about the further follow-up questionnaire surveying clinician attitudes and satisfaction with MBC, which showed clinician’s overall positive attitudes toward MBC, actual commitment in implementation, and their willingness to continuously incorporate MBC into their practice. On the other hand, staff reported significantly fewer facilitators than barriers. Clinicians intervene directly with patients in a formal care setting, whereas staff may receive more informal feedback from patients. Additionally, clinicians may be biased towards appreciating facilitators as they have more time to develop a relationship with patients as compared to staff and experience the external environmental expectations that they integrate MBC as a part of their standard care. Despite the lack of “readiness” for implementing this intervention illustrated by the lack of formalized MBC training at this institution, clinicians were knowledgeable or motivated to incorporate MBC based on their general attitudes toward MBC [46]. Clinician’s positive MBC attitudes were supported via quantitative analysis reflecting clinician’s understanding of the benefits of MBC and motivation for continuous MBC incorporation into their practice. Conversely, staff appeared to perceive more patient barriers related to their experiences providing a general explanation of MBC, guiding patients on the MFS including step by step procedures (i.e., help patients log-in, facilitate the completion of PROMs), and problem-solving when patients have difficulties (e.g., technical issues) in completing the measurements. As such, clinicians may see the positive effects of utilizing MBC with their patients that the staff does not, since staff are not directly involved in that aspect of care. It is possible that staff were unable to recognize the rationale or benefits of MBC as an EBP that ultimately improves intervention outcomes, as MBC was quickly introduced without formal training. This is hypothesized as a likely reason that staff expressed significantly more barriers than clinicians.

Furthermore, differences were observed between clinicians and staff responses according to the focus area (i.e., patient, clinician, staff, technology, institution) of barriers/facilitators. Clinicians reported patient-related barriers the most, followed by clinician-related and technology-related barriers. This data could reflect the clinician’s primary focus (i.e., patients) and their perception of primary obstacles when incorporating MBC in their usual care. These results are aligned with previous studies that investigated the barriers to MBC implementation on a different level, which showed multiple existing challenges on a patient and clinician level, though organizational and system level barriers were also identified [12]. Alternatively, staff reported technology-related barriers most frequently, followed by patient-related barriers, which could reflect the fact that staff often problem-solve both patients’ and clinicians’ technological issues. Regarding facilitators, clinicians reported patient-related facilitators and clinician-related facilitators (e.g., patients’ positive attitude on MBC, clinicians’ recognition of useful PROMs in their practice). Similarly, staff reported staff-related facilitators most often followed by institution-related facilitators. This data shows how each recipient group perceives different barriers or facilitators for implementing MBC, and provides insights considering that no known prior studies have identified these implementation barriers/facilitators from staff members who are directly facilitating patient completion of PROMs regularly. Table 6 summarizes the critical factors for implementation of MBC divided by each PRISM domain. These results implicate the need for further MBC training and re-implementation of MBC (i.e., ‘adaptable protocols and procedure’ elements in the implementation and sustainability infrastructure domain) in this adult outpatient psychiatry setting. By better understanding the recipient perspective, this data helps to inform holes in implementation and critical adoption barriers to increase fidelity and patient uptake. Previous research on MBC implementation was primarily focused on the training of clinicians, since these groups interact with patients the most [47]. However, in larger institutions, collaborative work among all recipients and solving the issues that each group faces could be the key to successful MBC incorporation to the usual care. Furthermore, the overall results demonstrate the need for organizational and system level changes to the external environment that can facilitate training which could include all recipients of MBC.

**Table 6:** Deconstructing the PRISM framework for MBC in adult ambulatory psychiatry based on the results of the virtual BPM

PRISM Component	Target	Recipients	Critical Points
**External Environment**	Adult ambulatory psychiatry - Department of Psychiatry and Behavioral Medicine’s (PBM) outpatient clinic in a US regional hospital	Clinicians	Agents of administration of measurement-based care (MBC) - Includes MD, NP, PhD, and LCSW
		Staff	Agents assisting implementation of MBC
		Patients	Receiving MBC by clinicians and provided help by staff
**Intervention**	Measurement-Based Care - Evidence-based practice (EBP) - Regularly evaluating patients’ mental and emotional state to facilitate treatment and inform clinical decision making - Utilizes patient-reported outcome measures (PROMs)	Clinicians	Implement MBC into their clinical practice - Utilize MBC to guide, track, and monitor treatment and patient’s symptoms - Review the results and have discussion with the patients - Incorporate PROMs results and inform clinical decision making
		Staff	Assisting administration - Prompt and guide patients to complete their PROMs - Problem-solve technical issues
		Patients	Participate in MBC - Complete PROMs regularly - Review their PROMs’ results and participate in collaborative evaluation with the clinician - Embrace information maximizing patient-centered care
**Implementation & Sustainability Infrastructure**	Focus Groups & Trainings - The present study conducted focus groups as a precursor to help develop and implement appropriate standardized MBC trainings - Brainwriting premortem method was utilized - Both clinicians and staff focus groups were conducted	Clinicians	Clinician focus groups resulted in 291 individual codes - Similar number of barriers and facilitators were identified - Main negative themes indicated clinician’s difficulty with patients (e.g., non-adherence), time burden, skepticism on PROMs’ usefulness, and lack of designated staff when utilizing MBC - Main positive themes indicated clinicians’ positive attitude toward MBC implementation into their practice
		Staff	Staff focus groups resulted in 97 codes - More barriers (67%) were identified than facilitators (24.7%) regarding MBC - Staff raised technology/virtual visit difficulties, patients’ negative attitude towards MBC and MFS, as well as chart integration issues
**Reach & Effectiveness**	Focus group results indicated current limitations and future directions for improved reach and effectiveness	Both clinicians and staff	Suggestions - Need for designated MBC/technology staff - Address patient non-adherence - Address ways to utilize MBC with certain type of patients (e.g., how to address high suicide risk patients, substance use/elderly patients are less likely to use MBC) - Plan for easy chart integration and visualization - Increase overall positive attitude and understanding of patients toward MBC
**Adoption**	Adopt MBC to adult ambulatory psychiatry	Clinician	Perceive the need for systematic and standardized training to better implement MBC into their clinical practice
		Staff	More resistant to implementing MBC policies and appear to have more negative perceptions of MBC and identify greater barriers to adoption
**Implementation**	Implement MBC to adult ambulatory psychiatry	Both clinicians and staff	Although MBC is already implemented, results of the BPM suggest that specific training is warranted and desirable for both staff and clinicians
**Maintenance**	Sustain the intervention (i.e., MBC) and systematically institutionalize MBC	All recipients of MBC	Implement routine trainings and assessments for implementation failures - Ensure systematic support and ongoing consultation by hospital to facilitate continuous utilization of MBC

Further analyses that identified the main themes from each focus group provided concrete themes for adapting the implementation process. Contrary to initial analyses, clinicians reported more negative themes than positive themes when individual responses were aggregated based on the specific content. Based on the review of negative themes, clinicians expressed patient-specific barriers, such as patient non-adherence, patients’ lack of understanding of MBC, and difficulty in utilizing MBC with certain types of patients. These results align with previous research identifying patients’ non-adherence issues [48] that could be resolved with clinician training on the ways to deliver and communicate with their patients [46]. Considering that the patient characteristics are crucial to understanding the recipients, identifying the patient’s burden, demands, and knowledge and beliefs would be one of the keys for successful implementation. In alignment with previous studies on the barriers of MBC, clinicians also reported an overall burden, time constraints, and skepticism on PROMs’ usefulness [49, 50]. Notably, clinicians participating in this study were institutionally required to engage their patients in MBC, and recipients still reported several difficulties that contribute to decreased utilization of MBC, and requested an improved implementation process, standardized training, and support for MBC. However, clinicians still reported valuable positive themes and benefits of MBC in their practice (i.e., reviewing PROMs results with patients; collaborative discussion). Considering that collaborative evaluation between clinician and patient is a key component of MBC [51], it should be emphasized continuously. Clinicians noted an overall positive view of MBC with specific evidence, including noticing certain patient types that are good candidates for MBC, and acknowledging the benefits of utilizing the PROMs results during their clinical practice. Similar to the previous analysis on barriers and facilitators, the staff group raised significantly higher issues and negative themes than positive themes or benefits of MBC. Since staff are the first-line personnel guiding and problem-solving patients confronting challenges on PROMs completion, these findings could imply setbacks and additional burdens when interacting with patients. This may also reflect limitations at the departmental level of support for staff and lack of feasible solutions for various issues that patients encounter in the process of participating in MBC. Related to the difficulties and issues that staff were facing, they reported a positive attitude toward and request for further in-depth MBC training and suggested their preferred training modality, which should be considered for future MBC training plans.

### Strengths and implications

The current study has several strengths. This study took a novel approach to integrate the implementation framework and BPM focus groups to infuse MBC into a medical setting. To date, the current study is the first to conduct virtual focus groups with both clinicians and staff to ensure effective MBC implementation in adult ambulatory psychiatry. The inclusion of front line staff stakeholders in the MBC process is often overlooked and provides a unique perspective on the shortcomings of MBC implementation. Comparing different perspectives provided practical implications for MBC implementation, considering the variability in standout themes. In addition, the inclusion of a focus group in the planning of this study’s implementation has illuminated a breakdown in understanding the roles recipients (i.e., clinicians and staff) play in the endorsement of a new intervention (i.e., MBC), and how that endorsement could potentially impact the implementation of MBC (i.e., actual usage with patients in a recommended way) and sustainability infrastructure, in the context of PRISM. It is critical that institutions recognize their MBC recipients’ perceptions, attitudes, and unique roles in MBC before the implementation process to increase the likelihood for sustainability success. In line with the PRISM framework, recognizing three or four domains (i.e., intervention, recipients, external environment, and/or implementation and sustainability infrastructure) with room for improvement will allow institutions to recognize which part of the implementation process needs changes or adjustments for successful implementation. Furthermore, the current study was conducted within an adult ambulatory psychiatry in a medical center, which faces different challenges compared to community clinics or psychology clinics that previous MBC implementation studies have focused on. Lastly, this study utilized mixed methods to grasp the whole picture of current MBC practices in this institution. Qualitative methods increased in-depth evaluation of participants’ perceptions, attitudes, and current status of their MBC implementation and utilization within the institution, and quantitative analysis pointed out the gap between the clinicians’ willingness towards MBC utilization versus their clinical practice in reality.

### Limitations and future directions

The current study featured a small sample size due to a limited participant pool to recruit employees engaged in the MBC process for focus groups, although the BPM method allowed for rapid idea generation, even with a smaller participant pool. Additionally, our findings are specific to a psychiatry and behavioral health clinic within a larger medical system which may not generalize to clinics where the clinicians and staff have no or minimal understanding of MBC, as the participants in this study were already engaging in MBC implementation after a brief, non standardized training. As such, the current study results may apply differently to the settings where the implementation level varies (e.g., prospective implementers; those who have been trained but not yet implemented; those who have implemented MBC as an EBP but are facing challenges). As qualitative coding still required a significant labor and time cost, it would be difficult to aggregate these codes rapidly for contemporaneous quality improvement projects. From a quantitative perspective, there was incomplete clinician and staff survey data. Additionally, the current study did not investigate patient perspectives on MBC, despite the patients being critical recipients of MBC. In future studies, investigators suggest collecting data from patients currently engaged in MBC to further enrich understanding of systematic strengths and faults. We also recommend an additional measurement be utilized to collect quantitative data reflective of staff attitudes.

## Conclusion

The present study highlights the necessity of recognizing relevant stakeholders from multidisciplinary healthcare teams, intervention, and the external environment of MBC implementation within the PRISM framework to ensure effective use of MBC and inform training efforts. The virtual focus groups utilized in this study played a crucial role in informing the development and re-implementation of the MBC training program and demonstrated that conducting virtual brainwriting premortem focus groups is feasible. It is possible that this was more feasible because it was online and allowed for greater participation between patient care. Participants offered invaluable observations, and the input from recipients who were already utilizing MBC with their patients informed the development of a targeted and tailored implementation program. Participants’ notion of some critical barriers informed the components that should be included in the training program. Specifically, future training is encouraged to include ways to improve patients’ adherence rate and increased understanding of MBC, ways to utilize MBC with various clientele, and systematic technological support. Moreover, participants expressed interest in and the necessity of the future training program, which could further support recipients and increase the possibility of high sustainability of the training program. Overall, the virtual BPM worked as a useful tool within this framework and has the potential to aid in the structural implementation of effective MBC in a psychiatric care setting. Future studies are needed to evaluate the effectiveness and sustainability of the MBC training program and implementation effort when addressing these barriers and facilitators noted by this study.

## Electronic supplementary material

Below is the link to the electronic supplementary material.

Supplementary Material 1

## Data Availability

The datasets generated and analyzed during the current study are not publicly available due to the size and rurality of the health care system and increased need for confidentiality, but are available from the corresponding author on reasonable request.

## Abbreviations

MBC: Measurement-Based Care
PROMs: Patient-Reported Outcome Measures
EBP: Evidence-Based Practice
MFS: Measurement-Feedback System, PRISM:Practical, Robust Implementation and Sustainability Model
RE-AIM: Reach, Effectiveness, Adoption, Implementation, and Maintenance of a program
BPM: Brainwriting Premortem Method
PBM: Department of Psychiatry and Behavioral Medicine
PRISM: Practical Robust Implementation and Sustainability Model
