# Supplementary material for: Improving measurement-based care implementation in adult ambulatory psychiatry: a virtual focus group interview with multidisciplinary healthcare professionals
Source: BMC Health Serv Res. 2023 Apr 26;23:408. doi: 10.1186/s12913-023-09202-3 (PMC10132409; doi:10.1186/s12913-023-09202-3)
Supplement: Supplementary file 1 — Supplementary Material 1 [file 12913_2023_9202_MOESM1_ESM.docx]

**Appendix A.** Clinician Focus Group Questions

| 1. As a clinician, what information is most valuable to you when seeing a patient? 2. Why do you use Patient Rated Outcome Measures (PROMs)? 3. Please share your thoughts about PROMs and using PROMs with patients. 4. In your experience, does the MBC system presented above reflect how the process actually happens? Please share how your experience with the process is similar or different. 5. What do you do with the information from the PROMs? 6. How do you use the information (e.g., scores, graph) from PROMs with your patients? 7. How do you provide feedback to your patients using PROMs? 8. Which PROMs do you use? 9. Which PROMs do you find useful/not useful? 10. What is the nature of the feedback you receive from your patients about the PROMs? 11. Do you notice a trend in the patients that value PROMs? Alternatively, are there patients that strongly oppose them? 12. Are there certain patients with whom you do not use PROMs? Why? 13. Do you believe the information patients share on the PROMs accurately reflects their concerns/symptoms? 14. For which patient groups/types do PROMs seem to be helping or not helping (in your opinion as a clinician)? Why? 15. Do you believe PROMs enhance your practice? How so? 16. Please share current barriers to effectively engaging in MBC, as well as current supports that have helped you engage in the process. 17. What additional support would improve or enhance your MBC practice? 18. Why will this training fail? (what could be problematic) 19. What training format would you prefer in order to learn more information about PROMs? (and about MBC?) 20. What else would you like to see in the training? 21. Any thoughts or concerns that were not addressed in this group about MBC? |
| --- |

**Appendix B.** Staff Focus Group Questions

| 1. Please share your understanding of the MBC process in your clinic. 2. Please share your thoughts about the MFS.\ 3. In your experience, does the MBC system presented above reflect how the process actually happens? Please share how your experience with the process is similar or different. 4. What is the nature of the feedback you receive from patients about the MBC process? 5. Do you notice a trend in the patients that value PROMs? Alternatively, are there patients that strongly oppose them? 6. What do you think would help patients fill out measures more often? 7. What is your role in the MBC process? 8. What kind of technical or practical support do you provide for the MFS? 9. Has having iPads available made the intake and measures process any faster or more efficient? 10. Do you feel comfortable assisting with the MBC process? How so? 11. Please share current barriers to effectively engaging in MBC, as well as current supports that have helped you engage in the process. 12. Do you think training would improve your ability to facilitate the MBC process? 13. Why will this training fail? (what could be problematic) 14. What training format would you prefer in order to learn more information about PROMs? (and about MBC?) 15. What else would you like to see in the training? 16. Any thoughts or concerns that were not addressed in this group about MBC? |
| --- |
